# A Phase 2a cohort expansion study to assess the safety, tolerability, and preliminary efficacy of CXD101 in patients with advanced solid-organ cancer expressing HR23B or lymphoma

**DOI:** 10.1186/s12885-021-08595-w

**Published:** 2021-07-23

**Authors:** Stephen W. Booth, Toby A. Eyre, John Whittaker, Leticia Campo, Lai Mun Wang, Elizabeth Soilleux, Daniel Royston, Gabrielle Rees, Murali Kesavan, Catherine Hildyard, Farasat Kazmi, Nick La Thangue, David Kerr, Mark R. Middleton, Graham P. Collins

**Affiliations:** 1 Department of Haematology, NIHR Oxford Biomedical Research Centre, Oxford University Hospitals, Oxford, UK; 2 Celleron Therapeutics Ltd, Oxford, UK; 3 Department of Oncology, University of Oxford, Oxford, UK; 4 Department of Cellular Pathology, Oxford University Hospitals, Oxford, UK; 5 Department of Pathology, University of Cambridge, Cambridge, UK; 6 Department of Haematology, Milton Keynes University Hospital, Milton Keynes, UK

**Keywords:** Histone deacetylase (HDAC), HR23B, Biomarker, Lymphoma

## Abstract

**Background:**

This Phase 2a dose expansion study was performed to assess the safety, tolerability and preliminary efficacy of the maximum tolerated dose of the oral histone de-acetylase (HDAC) inhibitor CXD101 in patients with relapsed / refractory lymphoma or advanced solid organ cancers and to assess HR23B protein expression by immunohistochemistry as a biomarker of HDAC inhibitor sensitivity.

**Methods:**

Patients with advanced solid-organ cancers with high HR23B expression or lymphomas received CXD101 at the recommended phase 2 dose (RP2D). Key exclusions: corrected QT > 450 ms, neutrophils < 1.5 × 10^9^/L, platelets < 75 × 10^9^/L, ECOG > 1. Baseline HR23B expression was assessed by immunohistochemistry.

**Results:**

Fifty-one patients enrolled between March 2014 and September 2019, 47 received CXD101 (19 solid-organ cancer, 28 lymphoma). Thirty-four patients received ≥80% RP2D. Baseline characteristics: median age 57.4 years, median prior lines 3, male sex 57%. The most common grade 3–4 adverse events were neutropenia (32%), thrombocytopenia (17%), anaemia (13%), and fatigue (9%) with no deaths on CXD101. No responses were seen in solid-organ cancers, with disease stabilisation in 36% or patients; the overall response rate in lymphoma was 17% with disease stabilisation in 52% of patients. Median progression-free survival was 1.2 months (95% confidence interval (CI) 1.2–5.4) in solid-organ cancers and 2.6 months (95%CI 1.2–5.6) in lymphomas. HR23B status did not predict response.

**Conclusions:**

CXD101 showed acceptable tolerability with efficacy seen in Hodgkin lymphoma, T-cell lymphoma and follicular lymphoma. Further studies assessing combination approaches are warranted.

**Trial registration:**

ClinicalTrials.gov identifier NCT01977638. Registered 07 November 2013.

**Supplementary Information:**

The online version contains supplementary material available at 10.1186/s12885-021-08595-w.

## Background

Epigenetic abnormalities are important in the pathogenesis of many cancers, amongst which histone modifications and associated changes in chromatin structure are some of the best described [1].

Histone de-acetylation is associated with a more closed chromatin configuration and silencing of tumour suppressor genes [2]. Currently licensed histone de-acetylase (HDAC) inhibitors are either non-selective across the four described classes of HDAC enzymes or have some bias for class 1 HDACs [3]. HDACs have also been described to have non-histone targets, for example deacetylation of the tumour suppressor p53 increases its activity, and other targets include heat shock protein 90 (HSP90) and nuclear factor kappa-light-chain-enhancer of activated B cells (NF-κB) [4, 5].

HDAC inhibitors have been studied across numerous cancer types and have gained regulatory approval in the United States in relapsed / refractory (R/R) cutaneous- and peripheral- T-cell lymphoma and in combination with proteasome inhibitors in myeloma. Overall response rates (ORRs) were 25–34% as single agent in T-cell lymphoma and 34.5% in combination with Bortezomib in myeloma [6], although single agent activity in myeloma was limited [7]. The pivotal Phase 2 studies of Romidepsin [8] and Belinostat [9] demonstrated a small number of patients with peripheral T-cell lymphoma had remarkably durable responses, but there are as yet no established biomarkers to predict response to HDAC inhibitors and potentially guide therapy [5]. Efficacy of HDAC inhibitors as monotherapy in solid organ malignancies has been limited [10, 11]. There are, however, numerous ongoing studies evaluating HDAC inhibitors in combination with other agents [5].

The HDAC inhibitors are generally well tolerated as monotherapy with the most common reported adverse events of grade 3 or more being thrombocytopenia, neutropenia, gastrointestinal symptoms and fatigue [8, 12, 13]. Electrocardiogram (ECG) QTc interval prolongation has been reported with HDAC inhibitors, warranting particular attention during safety assessment.

### CXD101

CXD101 is an investigational class 1-selective HDAC inhibitor. Following in vitro work demonstrating efficacy in colon, lung, non-Hodgkin lymphoma and myeloma cell lines, the Phase 1 dose escalation portion of this study established a recommended Phase 2 dose (RP2D) of 20 mg twice daily for 5 days of a 21-day cycle. Unlike the only approved HDAC inhibitor with class 1 selectivity, romidepsin, CXD101 has no class 2 activity and importantly is orally bioavailable [14, 15]. In contrast to the experimental HDAC inhibitor entinostat, which is orally bioavailable but inhibits both class 1 and class 4 HDACs, the half-life of CXD101 is considerably shorter (5–12 h) compared to 33–150 h) [16], reducing potential issues with accumulation and prolonged washout periods. It is hypothesised that in view of the tissue specificity of class 2 HDACs for the heart, smooth muscle, brain, liver and colon, that CXD101 may have reduced cardiac toxicity whilst preserving anti-tumour efficacy.

### HR23B

HR23B protein, also known as UV excision repair protein RAD23 homolog B, shuttles ubiquitinated cargo proteins to the proteasome and participates in nucleotide excision repair. A genome wide loss of function screen identified HR23B expression as a determinant of sensitivity to HDAC inhibitor induced apoptosis in an osteosarcoma cell line, and it is therefore a potential biomarker of HDAC inhibitor sensitivity [17]. The same authors demonstrated that reducing HR23B expression in vitro by short interfering RNA re-instated proteasome activity which had been suppressed in HDAC inhibitor treated cells, suggesting that HDAC inhibitors’ effect on the proteasome is mediated by HR23B. Immunohistochemical expression of HR23B has been reported to correlate with responses to HDAC inhibitors in cutaneous T-cell lymphoma, with high HR23B expression having a positive predictive value of 71.7% for clinical benefit (partial response or stable disease) [18]. A similar relationship between HR23B expression and response to HDAC inhibitors has been seen in hepatocellular carcinoma with higher levels of expression associated with higher rates of disease stabilisation [19].

Here we present final results of the Phase 2a expansion study evaluating the safety and efficacy CXD101 in lymphomas and advanced solid-organ malignancies expressing high levels of HR23B. We also assessed HR23B expression as a biomarker of response to HDAC inhibitors.

## Methods

### Study design

Detailed methods of the escalation portion of the study have been previously presented [20]. The trial was conducted at the Churchill Hospital, Oxford, United Kingdom. In brief, the study employed a single-arm 3 + 3 dose escalation to identify a maximum tolerated dose of CXD101, the highest dose at which fewer than 33% of patients experienced a pre-defined dose limiting toxicity. Patients were evaluated for safety on days 1, 2, 5 and 8 of cycles 1 and 2 and days 1 and a second time point of day 8–15 in subsequent cycles. Patients received CXD101 twice daily for days 1–5 of 21-day cycles until disease progression, unacceptable toxicity or withdrawal of consent. The primary end point of the expansion component of the study was assessment of the safety and toxicity of the RP2D. Pharmacokinetic data were collected and have been presented previously [20].

### Eligibility

Key inclusion criteria were: age > 18 years; with a measurable advanced malignant tumour [criteria of Cheson et al 2014] [21] for patients with lymphoma and Response Evaluation Criteria In Solid Tumors [RECIST; version 1.1] [22] for patients with solid organ malignancies; prior standard therapy; Eastern Cooperative Oncology Group (ECOG) performance status of 0 to 1; a life expectancy of ≥12 weeks; toxicity of previous treatment resolved to at least grade 1); adequate bone marrow, liver and renal function as defined by absolute neutrophil count ≥1.5 × 10^9^/L; platelets ≥75 × 10^9^/L; creatinine and bilirubin ≤1.5 x upper limit of normal; alanine aminotransferase or aspartate transaminase and alkaline phosphatase ≤2.5 x upper limit of normal.

In the expansion cohort patients with solid-organ cancers only were required to have high tumour expression of the HR23B by immunohistochemistry (IHC). This decision was taken in view of the lack of responses in unselected solid-organ cancer patients in the dose escalation cohort and emerging similar findings with other HDAC inhibitors. In contrast, responses were seen in patients with lymphoma, including those negative for HR23B and therefore this criterion was not applied to patients with lymphoma. Patients were excluded for: previous receipt of HDAC inhibitor; anticancer therapy within 28 days; mean corrected QT (QTc) > 450 milliseconds; positive serology for hepatitis B virus, hepatitis C virus or HIV; pregnancy or breast feeding; unwillingness to use contraception during and for 16 weeks after treatment with CXD101. Echocardiograms were not required at baseline. Staging was based on examination and computed tomography scan of the neck, chest, abdomen, and pelvis with unilateral bone marrow biopsy as indicated.

### Toxicity

Adverse events were categorised and graded according to the National Comprehensive Cancer Network Common Terminology Criteria for Adverse Events (CTCAE) version 4.03. Patients were assessed during screening and on cycle 2 day 15 by slit lamp and fundoscopy assessment. Triplicate electrocardiograms (ECGs) were performed at screening and all safety visits. Dosing was interrupted for development of QTc > 470 milliseconds until resolution to < 450 milliseconds and discontinued if QTc was increased by ≥60 milliseconds or to > 500 milliseconds. AEs are presented according to number and percentage of patients by worst grade experienced, and by the number and percentage of administered cycles affected to give an indication of the longitudinal persistence of toxicity over time and to facilitate comparison with published data from the escalation portion of the study [20, 23].

### Response evaluation

An important secondary end point of the study was preliminary assessment of the efficacy of CXD101 as monotherapy by ORR, defined as the rate of partial response (PR) or complete response (CR) as assessed in solid-organ cancers by RECIST v1.1 [22], and in lymphomas by the criteria of Cheson et al. [21]. ,Radiological assessment was by computed tomography performed at baseline and then every 2 treatment cycles. Patients without a progression event were censored at the time of their last assessment.

### Immunohistochemistry

Formalin-fixed, paraffin-embedded tissue was stained automatically with a BOND-MAX autostainer (Leica Microsystems Inc., Buffalo Grove, Illinois), using a commercial mouse monoclonal anti-HR23B antibody (BD.

Transduction Laboratories, Franklin Lakes, New Jersey). Two independent histopathologists blinded to patient outcome evaluated HR23B immunoreactivity in each sample compared to control colorectal carcinoma specimens of each intensity level. Scores were for combined nuclear and cytoplasmic expression (1 indicates < 5%, 2 indicates 25–50%, 3 indicates 50–75% and 4 indicates > 75%); and intensity (0 indicates negative, 1 indicates weak, 2 indicates moderate, and 3 indicates strong).

Within the expansion cohort, patients with solid-organ tumours were eligible according to positive expression (6–7 of 7 was considered positive). Archival or recent formalin-fixed, paraffin-embedded tissue was used when available.

### Statistical analysis

The data are presented descriptively as absolute values with percentages where relevant. Progression-free survival (PFS) was defined from the date of cycle 1 day 1 to progression or death and estimated using the method of Kaplan and Meier [24]. Median progression-free survival is presented with 95% confidence intervals (CI). Survival analyses were performed with Stata version 16.1 (Stata Corp., College Station, TX, USA).

## Results

### Baseline characteristics

A total of 51 patients were enrolled between 12 March 2014 and 05 September 2019. Four patients did not receive CXD101 because of deterioration in laboratory parameters causing them to be ineligible by the planned cycle 1 day 1. Thirty patients were treated in the escalation phase and 17 in the expansion phase. Data were censored 31 December 2020. Baseline characteristics of the 19 solid-organ cancer and 28 lymphoma patients treated are shown in Table 1. The whole population median age was 57.4 years (range 21.7–79.4), most of the cohort had received significant prior treatment (median 3 lines, range 0–10). Baseline tumour samples were available for 41 patients; HR23B expression by IHC was positive in 32 patients and negative 11 patients.

**Table 1:** Baseline characteristics

Characteristic	Solid tumour *N* = 19	Haematological malignancy *N* = 28	Combined *N* = 47
Sex			
Male	9	18	27
Female	10	10	20
Median age (range), years	59.2 (39.4–70.8)	49.0 (21.7–79.4)	53.1 (21.7–79.4)
Median no. of prior lines of systemic therapy (range)	2 (0–8)	4 (1–10)	3 (0–10)
ECOG performance status			
0	7	10	17
1	12	18	30
Histology	Colorectal 4	cHL 15	
	Lung 4	DLBCL 3	
	Upper GI 3	AITL 4	
	Cervix 2	PTCLNOS 2	
	Endometrial 1	Indolent B-cell NHL 3	
	Breast 1	GZL 1	
	Peritoneal 1		
	Neuroendocrine 1		
	Head and neck SCC 1		
	Meningioma 1		
Stage			
III	0	4	N/A
III or IIIS	2	6	
IV	17	18	
Baseline HR23B status (IHC)			
Positive (6–7)	15	17	32
Negative (0–5)	2	9	11
Not available	2	2	4

*GI* gastrointestinal, *ECOG* Eastern Cooperative Oncology Group, *cHL* classic Hodgkin lymphoma, *AITL* angio-immunoblastic T-cell lymphoma, *DLBCL* diffuse large B-cell lymphoma, *GZL* grey zone lymphoma, *HR23B* UV excision repair protein RAD23, *IHC* immunohistochemistry, *NHL* non-Hodgkin lymphoma, *PTCLNOS* Peripheral T-cell lymphoma not otherwise specified

### Safety

Details of the 3 + 3 dose escalation phase leading to a recommended phase 2 dose (RP2D) of 20 mg twice a day for 5 days of a 21-day cycle have been described elsewhere [20]. Neutropenia was the dose limiting toxicity. CXD101 was typically well tolerated. Details of treatment emergent adverse events (AE) s occurring in greater than or equal to 5% of patients are given in Table 2**.** A complete table of treatment emergent AEs is given in Supplementary Table 1. There were no deaths related to CXD101. 29 (60%) patients experienced grade 3–4 AEs, leading to a total of 19 admissions affecting 13 (28%) of the patients, 7 of these AEs (37%) were judged to be at least possibly related to CXD101. Grade 3–4 AEs leading to admission affected 17 (9%) of cycles. The majority of admissions (68%) related to infections. The most common grade 3–4 events were: neutropenia (15, 32%), thrombocytopenia (8, 17%), anaemia (6, 13%) and fatigue (4, 9%). Grade 3 or greater neutropenia was seen in 14% of cycles. Five episodes of febrile neutropenia occurred (3% of cycles), affecting a total of 4 (8.5%) of patients. Grade 3 or 4 infection was reported in 6 (13%) patients and occurred in 4% of cycles. Two (4%) patients had grade 3 or 4 QTc interval prolongation; one of these patients had a 12 year history of hypertension but neither had known cardiac disease. The most common grade 1–2 AEs were nausea 24 (51%), anaemia 21 (45%), fatigue 22 (47%), and vomiting 14 (30%) Any grade of nausea was reported in 22% of cycles and vomiting in 13%. At the time of the data cut off, 4 patients discontinued treatment for toxicity: 2 because of asymptomatic QT prolongation during the first cycle, 1 because of grade 4 fatigue in the 8 cycle and 1 because grade 4 thrombocytopenia during the second cycle, although simultaneous progression of T-cell lymphoma may have been a contributing factor in this case. Further to these, 3 patients discontinued because of investigator or patient decision and a single patient continued treatment, the remaining 41 stopped treatment for progressive disease.

**Table 2:** Treatment emergent adverse events occurring in ≥5% of patients

Adverse event term	All Grades		Grade 1 or 2				Grade 3 or 4			
	Patients	% patients (*N* = 47)	Patients	% patients (*N* = 47)	Number of cycles	% cycles (*N* = 194)	Patients	% patients (*N* = 47)	Number of cycles	% cycles (*N* = 194)
Blood and lymphatic system										
Anaemia	21	45%	15	32%	38	20%	6	13%	11	6%
Febrile Neutropenia	4	9%	0	0%	0	0%	4	9%	5	3%
Gastrointestinal disorders										
Abdominal Pain	10	21%	10	21%	15	8%	0	0%	0	0%
Diarrhoea	10	21%	8	17%	21	11%	2	4%	2	1%
Dyspepsia	3	6%	3	6%	3	2%	0	0%	0	0%
Mouth ulcer	3	6%	3	6%	4	2%	0	0%	0	0%
Nausea	24	51%	24	51%	42	22%	0	0%	0	0%
Vomiting	14	30%	14	30%	26	13%	0	0%	0	0%
General disorders										
Fatigue	22	47%	18	38%	53	27%	4	9%	4	2%
Fever	4	9%	4	9%	4	2%	0	0%	0	0%
Flu-like Symptoms	6	13%	6	13%	9	5%	0	0%	0	0%
Infections and infestations										
Bronchial Infection	4	9%	2	4%	7	4%	2	4%	2	1%
Lung infection	7	15%	4	9%	6	3%	3	6%	3	2%
Rhinitis infective	5	11%	5	11%	6	3%	0	0%	0	0%
UTI	7	15%	6	13%	7	4%	1	2%	1	1%
Investigations										
ECG QTc interval prolonged	16	34%	14	30%	26	13%	2	4%	2	1%
Neutropenia	22	47%	7	15%	37	19%	15	32%	28	14%
Thrombocytopenia	18	38%	10	21%	53	27%	8	17%	20	10%
Metabolism and nutritional disorders										
Anorexia	13	48%	13	28%	24	12%	0	0%	0	0%
Hypoalbuminemia	4	9%	4	9%	5	3%	0	0%	0	0%
Hypokalaemia	5	11%	5	11%	5	3%	1	2%	1	1%
Hypophosphatemia	3	6%	3	6%	4	2%	0	0%	0	0%
MSK and connective tissue										
Back Pain	3	6%	3	6%	3	2%	0	0%	0	0%
Muscle cramps	3	6%	3	6%	3	2%	0	0%	0	0%
Pain in extremity	4	9%	4	9%	4	2%	0	0%	0	0%
Nervous system disorders										
Headache	10	21%	8	17%	12	6%	0	0%	0	0%
Psychiatric disorders										
Depression	3	6%	2	4%	2	1%	0	0%	0	0%
Respiratory and thoracic disorders										
Cough	3	6%	3	6%	3	2%	0	0%	0	0%
Dyspnoea	4	9%	4	9%	6	3%	0	0%	0	0%

Adverse Events categorised and graded according to CTCAE v 4.03

*CTCAE* National Cancer Institute Common Terminology for Adverse Events, *ECG QTc* electrocardiogram corrected QT interval, *MSK* musculoskeletal, *UTI* urinary tract infection

### Efficacy

**Fig. 1:**
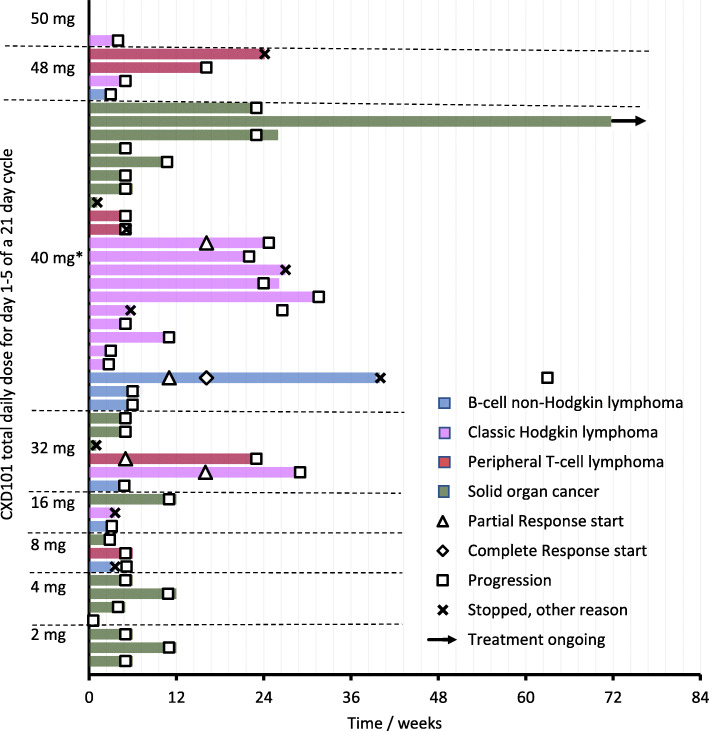
Swimmer plot of treatment duration by CXD101 dose and tumour histology * Recommended Phase 2 dose

Best response to CXD101 is shown in Table 3 for subjects dosed at ≥16 mg twice a day (80% of RP2D). There were no responses in the solid-organ cancer patients, although as shown in Fig. 1 there were 3 subjects treated for more than 6 cycles (4 months) without progression who derived clinical benefit (1 carcinoma of the cervix, 1 non-small cell lung carcinoma, and 1 peritoneal carcinoma): this last subject continues treatment after 24 cycles.

**Table 3:** Best response to CXD101 in patients dosed at CXD101 doses of ≥16 mg twice daily

Response	Solid organ, all HR23B positive *N* = 11	Lymphoma HR23B Negative *N* = 7	Lymphoma HR23B Positive *N* = 15	Lymphoma HR23B score not available *N* = 1	Lymphoma total *N* = 23	All patients *N* = 34
NE	2	1	0	0	3	5
PD	5	2	7	1	8	13
SD	4	2	6	0	8	12
PR	0	2	1	0	3	3
CR	0	0	1	0	1	1
ORR	0	29%	13%	0%	17%	12%

Responses shown according to RECIST v 1.1 for solid organ cancers and Cheson 2014 for lymphomas. HR23B status shown according to immunohistochemistry score on baseline tumour biopsy

*NE* not evaluable, *PD* progressive disease, *SD* stable disease, PR partial response, C*R* complete response, *ORR* Overall response rate (PR + CR)

**Fig. 2:**
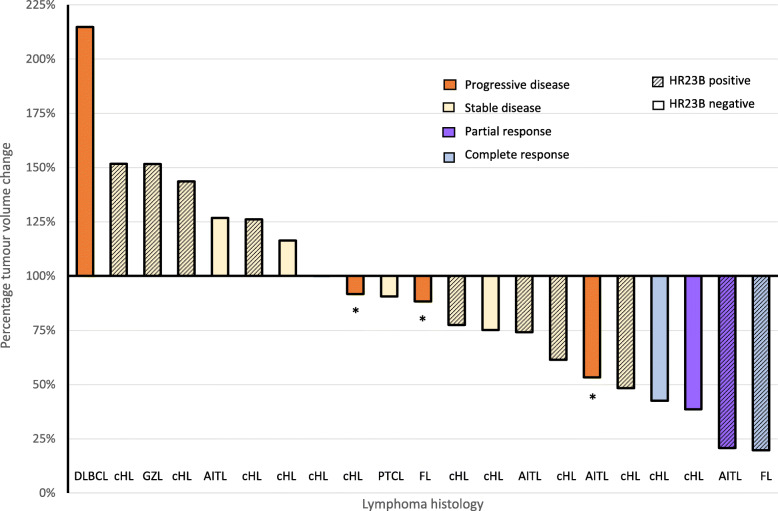
Best tumour responses in patients with lymphoma evaluable for response. Best tumour responses as assessed by sum of product diameters of target lesions in evaluable patients with lymphoma (in percentage) for CXD101 doses of ≥16 mg twice daily in patients shown according to baseline tumour HR23B status by immunohistochemistry and best disease response by Cheson et al. [21]. Two patients (1 FL and 1cHL) were not evaluable for objective response but clinically had a best response of progressive disease. * denotes patients with reduction in target lesions but clear progression of non-target lesions and / or new lesions. *cHL* classic Hodgkin lymphoma; *GZL* grey zone lymphoma; *AITL* angioimmunoblastic T-cell lymphoma; *PTCL* Peripheral T-cell lymphoma NOS; *FL* follicular lymphoma

In the subjects with lymphoma the ORR was 17% with 3 PRs and 1 CR (Table 3). The subject who attained a CR had follicular lymphoma, the 3 subjects with a PR had classic Hodgkin lymphoma (2 subjects) and angioimmunoblastic T-cell lymphoma (1 subject). The median duration of response was 6.3 months. A further 4 patients, all with classic Hodgkin lymphoma, were treated for more than 4 months and derived clinical benefit. As shown in Fig. 2, a reduction in tumour volume as assessed by the sum of products of diameters was seen in 6 of the 13 patients with a best response of stable disease.

Median PFS for the whole cohort was 1.2 months (95%CI 1.2–2.6), and for the solid organ patients 1.2 months (95%CI 1.2–5.4) and for the lymphoma patients 2.6 months (95%CI 1.2–5.6) Kaplan-Meier plots depicting the survival functions are shown in Supplementary Fig. 1.

### HR23B biomarker analysis

Baseline tumour biopsy samples for HR23B analysis by immunohistochemistry were available for 43 of the total 47 patients in the study (all the solid-organ cancer patients required an HR23b result for eligibility in the expansion phase). HR23B status was assessed in all the patients with available sample. Results showed good agreement between pathologists, with exact concordance in 31 of 44 samples; in the remaining 11 samples there were only 2 subjects (one diffuse large B-cell lymphoma (DLBCL) and one T-cell lymphoma) in which the discrepancy was by more than 1 point. Analysis of the dose escalation cohort has been presented previously [20]. Full HR23B data scoring data are given in Supplementary Table 2.

In the lymphoma patients treated with ≥16 mg twice daily baseline biopsies were available for 22 of the 23 patients and there was exact agreement between pathologists in 17 samples; in the remaining samples no discrepancy was by more than one point, and in no case was a there a discrepancy between pathologists as to whether the score was positive or negative.

Responses according to HR23B status are shown in Table 3. There was no clear correlation between HR23B status and probability of response. Fifteen of twenty-one (71%) evaluable subjects with lymphoma baseline tumour biopsies were positive for HR23B, amongst whom there was one CR and 1 PR. HR23B positive status therefore had a positive predictive value for objective response of 13% and for stable disease + objective response of 53%; and negative predictive value for objective response of 67% and for stable disease + objective response of 57%.

## Discussion

The study achieved the primary objective to investigate the safety, tolerability and dose limiting toxicity of CXD101 in patients with advanced malignancies, with a total of 36 patients treated at ≥80% of the R2PD.

Overall CXD101 was well tolerated, with no deaths on treatment. The adverse event profile is similar to that reported with other HDAC inhibitors. For example rates of ≥ grade 3 infection, thrombocytopenia, or, any grade of vomiting or diarrhoea are similar to data from Phase 2 studies of other HDAC inhibitors (vorinostat, romidepsin, belinostat, panobinostat): grade ≥ 3 infection 3–18%, grade ≥ 3 thrombocytopenia 2–26%, grade ≥ 3 anaemia 2–18%, any grade of vomiting 24–39% and any grade of diarrhoea 20–49% [7–9, 13, 25]. The rate of febrile neutropenia was low at 3% of cycles of CXD101. Clinically significant QT prolongation occurred in 2 patients. Although it is not possible to draw definite conclusions from the number of patients in this study regarding frequency of adverse events as compared to other HDAC inhibitors, QTc prolongation does occur with CXD101 and subsequent studies will provide further data to clarify this risk (e.g. NCT03993626). Despite the theoretical potential for retinal toxicity, this was not observed in the study.

Samples for baseline HR23B expression were available in more than 90% of patients. Scoring by IHC was reproducible with good agreement between pathologists. We did not observe a relationship between HR23B expression and objective response, or a combination of objective response and stable disease in the patients with lymphoma. This may have been a consequence of changes in HR23B expression between the biopsy date and starting trial treatment, given that the majority of biopsies were archival rather than fresh during screening and the stability of HR23B over time and with successive lines of therapy, which vary between patients, is poorly understood, as is also the case for many potential biomarkers. The heterogeneity of the lymphoma subtypes treated and their variation in histological growth patterns may also be a relevant factor and complicating the interpretation of an immunohistochemical scoring system as used in this study. On the basis of the current data HR23B on archival tissue is therefore not a biomarker for response to CXD101 in patients with unselected lymphomas [18]. Moreover, the principal mechanisms of CXD101 anti-tumour activity may be independent of HR23B and inhibition of proteasome function, for example through changes in histone structure or changes to acetylation states of non-histone proteins such as the tumour suppressor p53 [26–28]. Activation of immune mechanisms is also likely to be significant in CXD101’s action. In human colorectal cancer cell lines and murine colorectal cancer models CXD101 treatment has recently been shown by gene expression profiling to be associated with increased expression of genes associated with antigen processing and presentation, such as major histocompatibility antigen (MHC) class 1 and class 2 genes, as well as increased expression of genes involved pathways associated with natural killer cell mediated cytotoxicity [29]. An immune mechanism of action is supported by the finding in this study that lymphocyte and natural killer cell populations in the tumour microenvironment are altered by CXD101 and that efficacy of CXD101 is enhanced when combined with immune checkpoint inhibitors in murine models.

The lack of objective response in patients with solid organ malignancies is consistent with other published studies of HDAC inhibitors and the lack of licensed agents of this class for solid-organ cancers. Resistance to HDAC inhibitors as single agents is clearly an significant issue which remains incompletely understood, proposed mechanisms including increased expression of the cell cycle regulators B-cell lymphoma-2 or p21, and constitutive activation of NF-κB [5]. The previously published pharmacokinetic data demonstrate plasma levels well within the in vitro biologically active range at the RP2D, and there was no difference in plasma levels between responders and non-responders, making it unlikely that an alternative dose would deliver greater efficacy without unacceptable toxicity.

We have observed activity of CXD101 in relapsed / refractory lymphoma with an ORR of 17%. Responses were seen across the principle histological divisions (cHL, and both B-cell and T-cell NHL), despite a median of 4 prior lines of therapy. The responses proved reasonably durable at a median of 27 weeks, allowing one patient with cHL to be bridged directly to allogeneic haematopoietic stem cell transplant. Another patient with cHL with a best response of stable disease responded well to their next line of therapy with a platinum-based salvage chemotherapy regimen and also successfully underwent allogeneic-HSCT. The diversity of histologies and doses analysed treated in this initial Phase I/II study limits interpretation of this ORR, but to give some context, the reported ORR with single agent romidepsin or belinostat in R/R peripheral T-cell lymphoma was 25–26%; with panobinostat in cHL was 27%, and for vorinostat in follicular lymphoma was 49%.

Several other classes of agent have been proposed as rational combinations with HDAC inhibitors on the basis of in vitro data, including drugs targeting DNA repair mechanisms or the DNA damage response, immune checkpoint inhibitors, proteasome inhibitors and hypomethylating agents [5]. Data from several tumour types indicate HDAC inhibitors increase MHC class I expression, counteracting the immune evasion undertaken by many cancers, as well as increasing chemokine expression and T-cell recruitment to the tumour [29–34]. In mouse models HDAC inhibitors have been shown to restore sensitivity to PD-1 blockade in models of lymphoma [35]. CXD101 is currently undergoing evaluation in combination with nivolumab in patients with metastatic microsatellite-stable colorectal cancer, including assessment of HR23B alongside MHC I and II and PD-1 expression as potential biomarkers of response in this context. (ClinicalTrials.gov identifier: NCT03993626).

## Conclusions

CXD101 20 mg twice a day for 5 days of a 21 day cycle was tolerable and showed activity in lymphoma across a range of subtypes, but objective responses to single agent CXD101 were not seen in solid organ cancers. Further evaluation of the activity of CXD101 in specific tumour populations and in combination with checkpoint inhibitors, or potentially other agents, is warranted in order to better understand its optimal use and potential biomarkers of response.

## Supplementary Information

**Additional file 1: Table S1.** All treatment emergent adverse events categorised and graded according to CTCAE version 4.03. **Table S2.** HR23B score data for all treated patients. **Figure S1.** Progression-free Survival on CXD101.

## Data Availability

Data on which the study conclusions are based are provided in the supplementary data. Requests for further data should be submitted to the corresponding author.

## Abbreviations

AEs: Adverse events
AITL: Angio-immunoblastic T-cell lymphoma
cHL: Classic Hodgkin lymphoma
CR: Complete response
CTCAE: Common Terminology Criteria for Adverse Events
DLBCL: Diffuse Large B-cell lymphoma
DSR: Disease stabilisation rate
ECG: Electrocardiogram
ECOG: Eastern Cooperative Oncology Group performance status
GZL: Grey zone lymphoma
IHC: Immunohistochemistry
HDAC: Histone de-acetylase
HR23B: UV excision repair protein RAD23
RP2D: Recommended phase 2 dose
NF-κB: Nuclear factor kappa-light-chain-enhancer of activated B cells
NHL: Non-Hodgkin lymphoma
R/R: Relapsed / refractory
ORR: Overall response rate
PFS: Progression-free survival
PR: Partial response
PTCLNOS: Peripheral T-cell lymphoma not otherwise specified
