# Supplementary material for: A Phase 2a cohort expansion study to assess the safety, tolerability, and preliminary efficacy of CXD101 in patients with advanced solid-organ cancer expressing HR23B or lymphoma
Source: BMC Cancer. 2021 Jul 23;21:851. doi: 10.1186/s12885-021-08595-w (PMC8306282; doi:10.1186/s12885-021-08595-w)
Supplement: Supplementary file 1 — Additional file 1: Table S1. All treatment emergent adverse events categorised and graded according to CTCAE version 4.03. Table S2. HR23B score data for all treated patients. Figure S1. Progression-free Survival on CXD101. [file 12885_2021_8595_MOESM1_ESM.docx]

# **Supplementary information**:

| Supplementary Table 1: All treatment emergent adverse events categorised and graded according to CTCAE version 4.03 | | | | | | | | | | |  | | |  | | |  | | |
| --- | --- | --- | --- | --- | --- | --- | --- | --- | --- | --- | --- | --- | --- | --- | --- | --- | --- | --- | --- |
| Adverse event term | All Grades | | Grade 1 or 2 | | | | Grade 3 or 4 | | | |  | |  | | |  | | |  |
|  | Patients | % patients (*N* = 47) | Patients | % patients (*N* = 47) | Number of cycles | % cycles (*N* = 194) | Patients | % patients (*N* = 47) | Number of cycles | % cycles (*N* = 194) |  |  | | |  | | |  |  |
| I created Blood and lymphatic system | | | | | | | | | | |  | | |  | | |  | | |
| Anaemia | 21 | 45% | 15 | 32% | 38 | 20% | 6 | 13% | 11 | 6% |  |  | | |  | | |  |  |
| Febrile Neutropenia | 4 | 9% | 0 | 0% | 0 | 0% | 4 | 9% | 5 | 3% |  |  | | |  | | |  |  |
| Cardiac disorders | | | | | | | | | | |  | | |  | | |  | | |
| Myocardial Infarction | 0 | 0% | 0 | 0% | 0 | 0% | 1 | 2% | 1 | 1% |  |  | | |  | | |  |  |
| Palpitations | 1 | 2% | 1 | 2% | 1 | 1% | 0 | 0% | 0 | 0% |  |  | | |  | | |  |  |
| Ear and Labyrinth disorders | | | | | | | | | | |  | | |  | | |  | | |
| Ear pain | 1 | 2% | 1 | 2% | 1 | 1% | 0 | 0% | 0 | 0% |  |  | | |  | | |  |  |
| Tinnitus | 1 | 2% | 1 | 2% | 1 | 1% | 0 | 0% | 0 | 0% |  |  | | |  | | |  |  |
| Eye disorders | | | | | | | | | | |  | | |  | | |  | | |
| Blurred Vision | 1 | 2% | 1 | 2% | 1 | 1% | 0 | 0% | 0 | 0% |  |  | | |  | | |  |  |
| Stye | 1 | 2% | 1 | 2% | 1 | 1% | 0 | 0% | 0 | 0% |  |  | | |  | | |  |  |
| Gastrointestinal disorders | | | | | | | | | | |  | | |  | | |  | | |
| Abdominal Pain | 10 | 21% | 10 | 21% | 15 | 8% | 0 | 0% | 0 | 0% |  |  | | |  | | |  |  |
| Colonic fistula | 1 | 2% | 1 | 2% | 1 | 1% | 0 | 0% | 0 | 0% |  |  | | |  | | |  |  |
| Constipation | 2 | 4% | 2 | 4% | 4 | 2% | 0 | 0% | 0 | 0% |  |  | | |  | | |  |  |
| Diarrhoea | 10 | 21% | 8 | 17% | 21 | 11% | 2 | 4% | 2 | 1% |  |  | | |  | | |  |  |
| Dry mouth | 1 | 2% | 1 | 2% | 2 | 1% | 0 | 0% | 0 | 0% |  |  | | |  | | |  |  |
| Dysgeusia | 1 | 2% | 1 | 2% | 1 | 1% | 0 | 0% | 0 | 0% |  |  | | |  | | |  |  |
| Dyspepsia | 3 | 6% | 3 | 6% | 3 | 2% | 0 | 0% | 0 | 0% |  |  | | |  | | |  |  |
| Mouth ulcer | 4 | 9% | 3 | 6% | 4 | 2% | 0 | 0% | 0 | 0% |  |  | | |  | | |  |  |
| Nausea | 24 | 51% | 24 | 51% | 42 | 22% | 0 | 0% | 0 | 0% |  |  | | |  | | |  |  |
| Vomiting | 14 | 30% | 14 | 30% | 26 | 13% | 0 | 0% | 0 | 0% |  |  | | |  | | |  |  |
| General disorders | | | | | | | | | | |  | | |  | | |  | | |
| Chills | 1 | 2% | 1 | 2% | 1 | 1% | 0 | 0% | 0 | 0% |  |  | | |  | | |  |  |
| Fatigue | 22 | 47% | 18 | 38% | 53 | 27% | 4 | 9% | 4 | 2% |  |  | | |  | | |  |  |
| Fever | 4 | 9% | 4 | 9% | 4 | 2% | 0 | 0% | 0 | 0% |  |  | | |  | | |  |  |
| Flu-like Symptoms | 6 | 13% | 6 | 13% | 9 | 5% | 0 | 0% | 0 | 0% |  |  | | |  | | |  |  |
| Leg oedema | 1 | 2% | 1 | 2% | 2 | 1% | 0 | 0% | 0 | 0% |  |  | | |  | | |  |  |
| Night sweats | 2 | 4% | 2 | 4% | 2 | 1% | 0 | 0% | 0 | 0% |  |  | | |  | | |  |  |
| Non-cardiac chest pain | 2 | 4% | 1 | 2% | 1 | 1% | 1 | 2% | 1 | 1% |  |  | | |  | | |  |  |
| Hepatobiliary disorders | | | | | | | | | | |  | | |  | | |  | | |
| Cholecystitis | 1 | 2% | 0 | 0% | 0 | 0% | 1 | 2% | 2 | 1% |  |  | | |  | | |  |  |
| Infections and infestations | | | | | | | | | | |  | | |  | | |  | | |
| Appendicitis | 1 | 2% | 0 | 0% | 0 | 0% | 1 | 2% | 1 | 1% |  |  | | |  | | |  |  |
| Bronchial Infection | 4 | 9% | 2 | 4% | 7 | 4% | 2 | 4% | 2 | 1% |  |  | | |  | | |  |  |
| Catheter related infection | 1 | 2% | 1 | 2% | 1 | 1% | 0 | 0% | 0 | 0% |  |  | | |  | | |  |  |
| Lung infection | 7 | 15% | 4 | 9% | 6 | 3% | 3 | 6% | 3 | 2% |  |  | | |  | | |  |  |
| Oral candida | 1 | 2% | 1 | 2% | 1 | 1% | 0 | 0% | 0 | 0% |  |  | | |  | | |  |  |
| Rhinitis infective | 5 | 11% | 5 | 11% | 6 | 3% | 0 | 0% | 0 | 0% |  |  | | |  | | |  |  |
| Sepsis | 1 | 2% | 0 | 0% | 0 | 0% | 1 | 2% | 1 | 1% |  |  | | |  | | |  |  |
| Sinusitis | 1 | 2% | 1 | 2% | 1 | 1% | 0 | 0% | 0 | 0% |  |  | | |  | | |  |  |
| Adverse event term | All Grades | | Grade 1 or 2 | | | | Grade 3 or 4 | | | |  |  | | |  | | |  |  |
|  | Events | % patients (*N* = 47) | Events | % patients (*N* = 47) | Number of cycles | % cycles (*N* = 194) | Events | % patients (*N* = 47) | Number of cycles | % cycles (*N* = 194) |  |  | | |  | | |  |  |
| Skin infection | 1 | 2% | 1 | 2% | 2 | 1% | 0 | 0% | 0 | 0% |  |  | | |  | | |  |  |
| Urinary Tract Infection | 7 | 15% | 6 | 13% | 7 | 4% | 1 | 2% | 1 | 1% |  |  | | |  | | |  |  |
| Injury, poisoning and procedural complications | | | | | | | | | | |  | | |  | | |  | | |
| Eye Injury | 1 | 2% | 1 | 2% | 1 | 1% | 0 | 0% | 0 | 0% |  |  | | |  | | |  |  |
| Fall | 1 | 2% | 1 | 2% | 2 | 1% | 0 | 0% | 0 | 0% |  |  | | |  | | |  |  |
| Investigations | | | | | | | | | | |  | | |  | | |  | | |
| ALP increased | 1 | 2% | 1 | 2% | 3 | 2% | 0 | 0% | 0 | 0% |  |  | | |  | | |  |  |
| ALT increased | 1 | 2% | 1 | 2% | 1 | 1% | 0 | 0% | 0 | 0% |  |  | | |  | | |  |  |
| Blood bilirubin increased | 2 | 4% | 2 | 4% | 3 | 2% | 0 | 0% | 0 | 0% |  |  | | |  | | |  |  |
| ECG QTc interval prolonged | 16 | 34% | 14 | 30% | 26 | 13% | 2 | 4% | 2 | 1% |  |  | | |  | | |  |  |
| Hypophosphatemia | 1 | 2% | 0 | 0% | 0 | 0% | 1 | 2% | 1 | 1% |  |  | | |  | | |  |  |
| Leucopenia | 2 | 4% | 1 | 2% | 11 | 6% | 6 | 13% | 8 | 4% |  |  | | |  | | |  |  |
| Lymphocyte count decreased | 1 | 2% | 0 | 0% | 1 | 1% | 2 | 4% | 3 | 2% |  |  | | |  | | |  |  |
| Neutropenia | 12 | 26% | 7 | 15% | 37 | 19% | 15 | 32% | 28 | 14% |  |  | | |  | | |  |  |
| Thrombocytopenia | 15 | 32% | 10 | 21% | 53 | 27% | 8 | 17% | 20 | 10% |  |  | | |  | | |  |  |
| Weight Loss | 4 | 9% | 2 | 4% | 2 | 1% | 0 | 0% | 0 | 0% |  |  | | |  | | |  |  |
| Metabolism and nutritional disorders | | | | | | | | | | |  | | |  | | |  | | |
| Anorexia | 20 | 43% | 13 | 28% | 24 | 12% | 0 | 0% | 0 | 0% |  |  | | |  | | |  |  |
| Hypoalbuminemia | 8 | 17% | 4 | 9% | 5 | 3% | 0 | 0% | 0 | 0% |  |  | | |  | | |  |  |
| Hypoalbuminemia | 1 | 2% | 0 | 0% | 0 | 0% | 1 | 2% | 1 | 1% |  |  | | |  | | |  |  |
| Hypokalaemia | 6 | 13% | 5 | 11% | 5 | 3% | 1 | 2% | 1 | 1% |  |  | | |  | | |  |  |
| Hyponatraemia | 1 | 2% | 1 | 2% | 1 | 1% | 1 | 2% | 1 | 1% |  |  | | |  | | |  |  |
| Hypophosphatemia | 6 | 13% | 3 | 6% | 4 | 2% | 0 | 0% | 0 | 0% |  |  | | |  | | |  |  |
| MSK and connective tissue | | | | | | | | | | |  | | |  | | |  | | |
| Arthralgia | 1 | 2% | 1 | 2% | 1 | 1% | 0 | 0% | 0 | 0% |  |  | | |  | | |  |  |
| Back Pain | 3 | 6% | 3 | 6% | 3 | 2% | 0 | 0% | 0 | 0% |  |  | | |  | | |  |  |
| Bone Pain | 1 | 2% | 1 | 2% | 1 | 1% | 0 | 0% | 0 | 0% |  |  | | |  | | |  |  |
| Flank pain | 1 | 2% | 1 | 2% | 1 | 1% | 0 | 0% | 0 | 0% |  |  | | |  | | |  |  |
| Hip pain | 1 | 2% | 1 | 2% | 1 | 1% | 0 | 0% | 0 | 0% |  |  | | |  | | |  |  |
| Muscle cramps | 3 | 6% | 3 | 6% | 3 | 2% | 0 | 0% | 0 | 0% |  |  | | |  | | |  |  |
| Myalgia | 1 | 2% | 1 | 2% | 1 | 1% | 0 | 0% | 0 | 0% |  |  | | |  | | |  |  |
| Neck Pain | 1 | 2% | 1 | 2% | 1 | 1% | 0 | 0% | 0 | 0% |  |  | | |  | | |  |  |
| Pain in extremity | 4 | 9% | 4 | 9% | 4 | 2% | 0 | 0% | 0 | 0% |  |  | | |  | | |  |  |
| Nervous system disorders | | | | | | | | | | |  | | |  | | |  | | |
| Cranial Nerve Palsy | 1 | 2% | 0 | 0% | 1 | 1% | 1 | 2% | 1 | 1% |  |  | | |  | | |  |  |
| Headache | 8 | 17% | 8 | 17% | 12 | 6% | 0 | 0% | 0 | 0% |  |  | | |  | | |  |  |
| Lethargy | 2 | 4% | 1 | 2% | 1 | 1% | 0 | 0% | 0 | 0% |  |  | | |  | | |  |  |
| Peripheral Sensory Neuropathy | 1 | 2% | 1 | 2% | 2 | 1% | 0 | 0% | 0 | 0% |  |  | | |  | | |  |  |
| Vasovagal Reaction | 1 | 2% | 0 | 0% | 0 | 0% | 1 | 2% | 1 | 1% |  |  | | |  | | |  |  |
| Psychiatric disorders | | | | | | | | | | |  |  | | |  | | |  |  |
| Depression | 3 | 6% | 2 | 4% | 2 | 1% | 0 | 0% | 0 | 0% |  |  | | |  | | |  |  |
| Hallucinations | 1 | 2% | 1 | 2% | 1 | 1% | 0 | 0% | 0 | 0% |  |  | | |  | | |  |  |
| Insomnia | 1 | 2% | 1 | 2% | 1 | 1% | 0 | 0% | 0 | 0% |  |  | | |  | | |  |  |
| Renal and urinary disorders | | | | | | | | | | |  | | |  | | |  | | |
| Acute Kidney Injury | 1 | 2% | 1 | 2% | 1 | 1% | 0 | 0% | 0 | 0% |  |  | | |  | | |  |  |
| Urinary tract pain | 1 | 2% | 1 | 2% | 2 | 1% | 0 | 0% | 0 | 0% |  |  | | |  | | |  |  |
|  |  | | |  | | | |  | | |  |  | | |  | | |  |  |
| Adverse event term | All Grades | | | Grade 1 or 2 | | | | Grade 3 or 4 | | |  |  | | |  | | |  |  |
|  | Events | % patients (*N* = 47) | Events | % patients (*N* = 47) | Number of cycles | % cycles (*N* = 194) | Events | % patients (*N* = 47) | Number of cycles | % cycles (*N* = 194) |  |  | | |  | | |  |  |
| Reproductive system and breast disorders | | | | | | | | | | |  |  | | |  | | |  |  |
| Erectile Dysfunction | 1 | 2% | 1 | 2% | 1 | 1% | 0 | 0% | 0 | 0% |  |  | | |  | | |  |  |
| Respiratory and thoracic disorders | | | | | | | | | | |  | | |  | | |  | | |
| Cough | 3 | 6% | 3 | 6% | 3 | 2% | 0 | 0% | 0 | 0% |  |  | | |  | | |  |  |
| Dyspnoea | 4 | 9% | 4 | 9% | 6 | 3% | 0 | 0% | 0 | 0% |  |  | | |  | | |  |  |
| Pleural effusion | 1 | 2% | 1 | 2% | 1 | 1% | 0 | 0% | 0 | 0% |  |  | | |  | | |  |  |
| Skin and subcutaneous disorders | | | | | | | | | | |  | | |  | | |  | | |
| Alopecia | 1 | 2% | 1 | 2% | 1 | 1% | 0 | 0% | 0 | 0% |  |  | | |  | | |  |  |
| Herpes re-activation | 1 | 2% | 1 | 2% | 1 | 1% | 0 | 0% | 0 | 0% |  |  | | |  | | |  |  |
| Papulopustular rash | 1 | 2% | 1 | 2% | 1 | 1% | 0 | 0% | 0 | 0% |  |  | | |  | | |  |  |
| Pruritus | 2 | 4% | 2 | 4% | 2 | 1% | 0 | 0% | 0 | 0% |  |  | | |  | | |  |  |
| Skin Rash | 2 | 4% | 2 | 4% | 2 | 1% | 0 | 0% | 0 | 0% |  |  | | |  | | |  |  |
| Vascular disorders | | | | | | | | | | |  | | |  | | |  | | |
| Flushing | 1 | 2% | 1 | 2% | 1 | 1% | 0 | 0% | 0 | 0% |  |  | | |  | | |  |  |
| Superior Vena Cava Syndrome | 1 | 2% | 0 | 0% | 0 | 0% | 1 | 2% | 1 | 1% |  |  | | |  | | |  |  |
| Thromboembolic event | 2 | 4% | 2 | 4% | 2 | 1% | 0 | 0% | 0 | 0% |  |  | | |  | | |  |  |

*ALP* alkaline phosphatase; *ALT* alanine aminotransferase*; CTCAE* National Cancer Institute Common Terminology for Adverse Events; *ECG QTc* electrocardiogram corrected QT interval; *MSK* musculoskeletal.

# Supplementary Table 2: HR23B score data for all treated patients.

| **Trial ID** | **Tumour Histology** | **HR23B Score Pathologist 1** | **HR23B Score Pathologist 2** | **Mean Score** | **Exact Concordance** |
| --- | --- | --- | --- | --- | --- |
| 1 | Lung | 6 | 5 | 5.5 | No |
| 2 | Colorectal | N/A | N/A | N/A | N/A |
| 3 | Breast | 7 | 7 | 7 | Yes |
| 4 | DLBCL | 6 | 6 | 6 | Yes |
| 5 | SCC | 6 | 6 | 6 | Yes |
| 6 | Pancreas | 7 | 6 | 6.5 | No |
| 7 | Endometrial | 5 | 5 | 5 | Yes |
| 8 | T cell | 5 | 7 | 6 | No |
| 9 | LPL | 3 | 3 | 3 | Yes |
| 10 | Lung | N/A | N/A | N/A | N/A |
| 11 | Colorectal | 7 | 6 | 6.5 | No |
| 13 | HL | N/A | N/A | N/A | N/A |
| 15 | DLBCL | 4 | 2 | 3 | No |
| 16 | HL | 5 | 5 | 5 | Yes |
| 17 | Cervical | 6 | 6 | 6 | Yes |
| 18 | Ovarian | 7 | 7 | 7 | Yes |
| 19 | DLBCL | 7 | 7 | 7 | Yes |
| 20 | AITL | 6 | 6 | 6 | Yes |
| 21 | Colorectal | 7 | 7 | 7 | Yes |
| 22 | HL | 6 | 7 | 6.5 | No |
| 23 | HL | 5 | 4 | 4.5 | No |
| 24 | HL | 6 | 7 | 6.5 | No |
| 25 | AITL | 6 | 6 | 6 | Yes |
| 26 | AITL | 5 | 5 | 5 | Yes |
| 27 | Cervical | 7 | 7 | 7 | Yes |
| 28 | HL | 6 | 6 | 6 | Yes |
| 29 | PTCLNOS | 4 | 5 | 4.5 | No |
| 30 | GZL | 7 | 7 | 7 | Yes |
| 31 | AITL | 6 | 6 | 6 | Yes |
| 32 | HL | 6 | 6 | 6 | Yes |
| 34 | HL | 6 | 6 | 6 | Yes |
| 35 | FL | 6 | 6 | 6 | Yes |
| 36 | HL | 6 | 7 | 6.5 | No |
| 37 | Oesophageal | 7 | 7 | 7 | Yes |
| 38 | HL | 5 | 5 | 5 | Yes |
| 39 | Anaplastic Meningioma | 7 | 7 | 7 | Yes |
| 40 | FL | 7 | 7 | 7 | Yes |
| 41 | Thyroid Carcinoma | 6 | 6 | 6 | Yes |
| 42 | HL | 6 | 6 | 6 | Yes |
| 43 | Colorectal Carcinoma | 7 | 6 | 6.5 | No |
| 44 | HL | N/A | N/A | N/A | N/A |
| 45 | HL | 4 | 4 | 4 | Yes |
| 46 | Oesophageal Carcinoma | 7 | 6 | 6.5 | No |
| 47 | FL | 0 | 0 | 0 | Yes |
| 48 | Non-small cell Lung Carcinoma | 7 | 7 | 7 | Yes |
| 50 | Carcinoma of Unknown Primary | 7 | 7 | 7 | Yes |
| 51 | HL | 7 | 7 | 7 | Yes |

*N/A* not applicable. *cHL* classic Hodgkin lymphoma; *GZL* grey zone lymphoma; *AITL* angioimmunoblastic T-cell lymphoma; *PTCL* Peripheral T-cell lymphoma NOS; *FL* follicular lymphoma; *LPL* lymphoplasmacytic lymphoma; *SCC* squamous cell carcinoma.

# Supplementary Figure 1. Progression-free Survival on CXD101


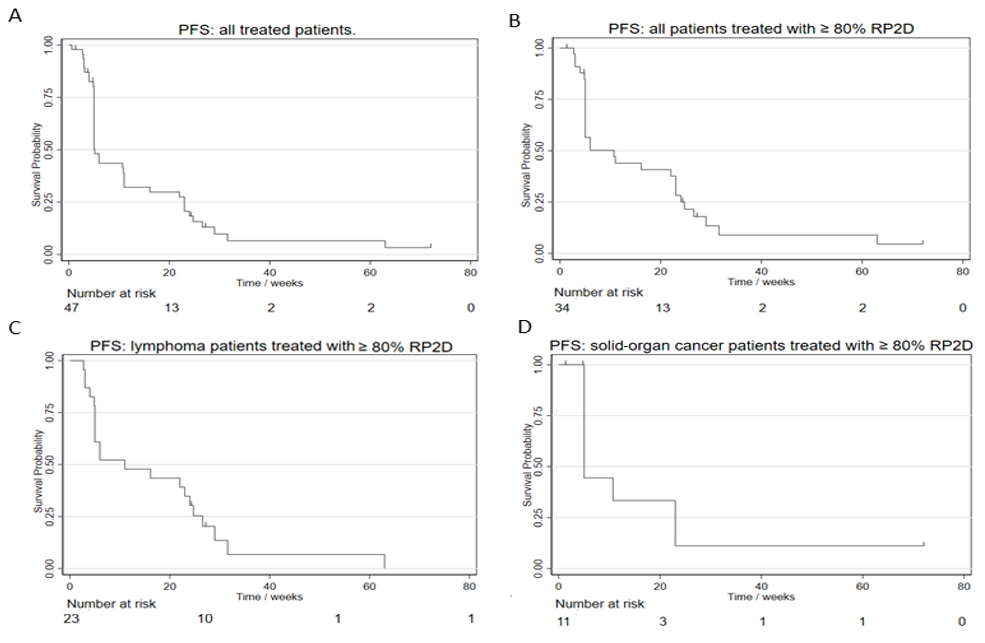


Progression-free survival (PFS) on CXD101 of a) all treated patients, b) all patients treated with ≥80% recommended Phae 2 Dose (RP2D), c) all patients with lymphoma treated with ≥80% RP2D, d) all patients with solid-organ cancer treated with ≥80% RP2D.
